# Case report: Noonan syndrome with protein-losing enteropathy

**DOI:** 10.3389/fgene.2023.1237821

**Published:** 2023-09-27

**Authors:** Yang Ou, Jun-Chao Yuan, Yao Zheng, Jin-Man Zhang, Tian He, Zhi Liang, Yi-Kun Zhou

**Affiliations:** ^1^ Department of Endocrinology and Metabolism, First People’s Hospital of Yunnan Province, The Kunhua Affiliated Hospital of Kunming University of Science and Technology, Kunming, China; ^2^ Department of Medical Genetics, First People’s Hospital of Yunnan Province, The Kunhua Affiliated Hospital of Kunming University of Science and Technology, Kunming, China; ^3^ Department of Gastroenterology, First People’s Hospital of Yunnan Province, The Kunhua Affiliated Hospital of Kunming University of Science and Technology, Kunming, China; ^4^ Department of Information Center, First People’s Hospital of Yunnan Province, The Kunhua Affiliated Hospital of Kunming University of Science and Technology, Kunming, China

**Keywords:** Noonan syndrome, lymphatic abnormalities, protein-losing enteropathy, PTPN11, intestinal lymphatic vessels

## Abstract

**Background:** Noonan syndrome (NS) is characterized by typical facial features, short stature, congenital heart defects and other comorbidities. Lymphedema and chylous pleural effusions are also common in NS, but protein-losing enteropathy (PLE) is rarely reported.

**Case presentation:** We present the case of a 19-year-old Chinese woman presenting with PLE. Small intestine biopsy showed obvious expansion of lymphatic vessels. The gene mutation results of the patient indicated a c.184T>G missense mutation (p.Tyr62Asp) in the PTPN11 gene (NM_002834.3).

**Conclusion:** NS accompanied by PLE is not common, but hypoproteinemia attributable to PLE may be more common in patients with NS than previously thought. It remains uncertain whether mutation of the PTPN11 gene is related to PLE, indicating that further research is needed.

## Introduction

Noonan and Elimke first described a disease similar to Turner`s syndrome in 1963 in patients with a female or male phenotype, right-sided congenital heart diseases and a normal karyotype (Noonan, 1968). Henceforth, this type of disease with the same clinical manifestations is called Noonan syndrome (NS), which is characterized by typical facial features (ptosis and low-set posteriorly rotated ears), short stature, congenital heart defects, cryptorchidism in males, webbed neck, mild intellectual deficit, lymphatic dysplasia and other comorbidities (van der Burgt, 2007). The incidence of NS has been estimated to be between 1:1,000 and 1:2,500 live births (van der Burgt, 2007). NS is caused by germline pathogenic variant genes in the Ras/mitogen activated protein kinase (MAPK) signal transduction pathway (Sleutjes et al., 2022). At present, mutations of 10 genes, including protein tyrosine phosphatase non-receptor type 11 (PTPN11), son of sevenless homolog 1 (SOS1), Raf-1 proto-oncogene, serine/threonine kinase (RAF1), neuroblastoma RAS viral oncogene homolog (NRAS) and soc-2 suppressor of clear homolog (SHOC2), have been found (Tartaglia et al., 2007; Tartaglia et al., 2010; Kouz et al., 2016). Approximately 58% of NS cases are caused by missense mutations in the PTPN11 gene on chromosome 12 (Chinton et al., 2019). Different pathogenic mutations usually determine the manifestation of NS.

Lymphedema, mainly due to hypoplasia or dysplasia of lymphatic vessels, is also a clinical manifestation of NS. The estimated lifetime prevalence of lymphatic abnormalities in NS patients is 20%, including lymphedema, chylothorax and pulmonary lymphangiectasia (Tsang et al., 2000; Romano et al., 2010; Kouz et al., 2016; Biko et al., 2019). The most common lymphatic abnormality is lymphedema, which usually appears at birth and disappears in early childhood (Bloomfield et al., 1997; Ho et al., 2003). Lymphatic dysplasia can also be diagnosed by ultrasound in the prenatal phase based on increased neck translucency, cystic hygroma, pleural effusion and cervical lymphatic sac expansion, which all suggest the diagnosis of NS (Stuurman et al., 2019). However, lymphatic dysplasia may also occur in late childhood and adulthood (Romano et al., 2010). Severe lymphatic diseases mainly include chylothorax and protein-losing enteropathy (PLE) (Dori et al., 2020). Although lymphedema and chylous pleural effusions are common in NS (Lanning et al., 1978), the incidence of PLE is relatively low. PLE typically manifests as either syndromic or non-syndromic and is commonly associated with primary intestinal lymphangiectasia. Existing research has identified specific gene mutations that are linked to PLE (Fang et al., 2000; Van Balkom et al., 2002; Irrthum et al., 2003; Fotiou et al., 2015; van Rijn et al., 2018), including mutations in collagen and calcium binding EGF domains 1 (CCEB1), FAT tumor suppressor homolog 4 (FAT4), Piezo type mechanosensitive ion channel component 1 (PIEZO1), forkhead box C2 (FOXC2) and complement decay-accelerating factor (CD55). However, the association between PLE and specific gene mutations in NS has not been determined.

Here, we present the case of a 19-year-old Chinese woman presenting with severe edema and hypoproteinemia.

## Case description

A 19-year-old woman was hospitalized due to progressive lower limb edema and oliguria for 2 weeks. Before admission, the patient’s laboratory results were as follows: albumin, 20.2 g/L (normal range of 40–55 g/L); calcium, 0.93 mmol/L (normal range of 2.11–2.52 mmol/L); potassium, 2.94 mmol/L (normal range of 3.5–5.3 mmol/L); blood routine hemoglobin, 97 g (normal range of 115–150 g); and urine samples showed positive urine protein. However, no definitive diagnosis was made. Only oral potassium chloride supplement was given, and the patient later presented to our hospital. The patient underwent surgical treatment for ptosis at age 11. She had menstruation at 12 years of age, and her menstrual cycle has been normal since then. There were no similar cases in the family, and no other family history required attention.

Physical examination showed the typical facial and skeletal abnormalities of NS as follows: height of 140 cm; weight of 48 kg, right ptosis, eye distance widened; low posterior hairline; low-set and posteriorly rotated ears; webbing of the neck; and high jaw bow. A grade 2–3 systolic blowing murmur was heard in the second intercostal space at the left edge of the sternum. Edema was generalized with severe pitting edema of both lower limbs.

## Results

The laboratory study results can be found Table 1.

**TABLE 1:** The laboratory test results.

Examination items	Results	Normal range
white blood cell count	10.48 × 10^9^/L	3.5–9.5 × 10^9^/L
absolute lymphocyte count	0.21 × 10^9^/L	1.1–3.2 × 10^9^/L
hemoglobin	88 g	115–150 g
AST	134 U/L	13–55 U/L
ALT	45 U/L	7–40 U/L
total protein	29.6 g/L	65–85 g/L
albumin	16 g/L	40–55 g/L
corrected calcium	1.22 mmol/L	2.11–2.52 mmol/L
potassium	3.2 mmol/L	3.5–5.3 mmol/L
parathyroid hormone	202 pg/mL	15–68.3 pg/mL
synchronous 24 h urine calcium	0.14 mmol/24 h	—
total 25-hydroxyvitamin D	˂3.00 ng/mL	˃30 ng/mL
1,25-dihydroxyvitamin D3	36.47 pg/mL	18–78 pg/mL
IgG	2.47 g/L	8.6–17.4 g/L
IgA	0.36 g/L	1.0–4.2 g/L
IgM	0.59 g/L	0.5–2.8 g/L

After calcium supplementation, the parathyroid hormone returned to normal (54.80 pg/mL), and pseudohypoparathyroidism was temporarily excluded. The arginine plus levodopa growth hormone stimulation test showed no growth hormone deficiency (fasting, 3.070 ng/mL; 0.5 h, 4.750 ng/mL; 1 h, 5.370 ng/mL; 1.5 h, 5.900; and 2.0 h, 13.530 ng/mL). The antibody related to celiac disease was normal. The bone marrow puncture results showed proliferative anemia, potential iron deficiency anemia, nucleated cell proliferation, granulocyte proliferation, normal erythron, normal megakaryocytes and no abnormal cells. The left wrist bone age radiograph provided the following bone ages: life age was 19 years and 5 months; R series bone age was 15 years and 10 months, which was 3 years and 7 months younger than the actual age of the patient; and C series bone age was 11 years and 0 months, which was 8 years and 5 months younger than the actual age of the patient. The cardiac ultrasound indicated congenital heart disease with an atrial septal defect (the defect opening of the left to right shunt in the center of the secondary foramen was approximately 0.62 cm wide and 0.56 cm wide, respectively). The plain computed tomography (CT) chest scan indicated a small amount of pleural effusion on both sides, and the heart border was slightly larger. The plain CT scan of the abdomen and pelvis indicated increased and blurred density of the peritoneal mesangium and omentum, and the surrounding fat was turbid, especially at the root of the mesangium. Scattered lymph nodes in the mesangium were observed, and the left anterior renal fascia was slightly thickened.

**FIGURE 1:**
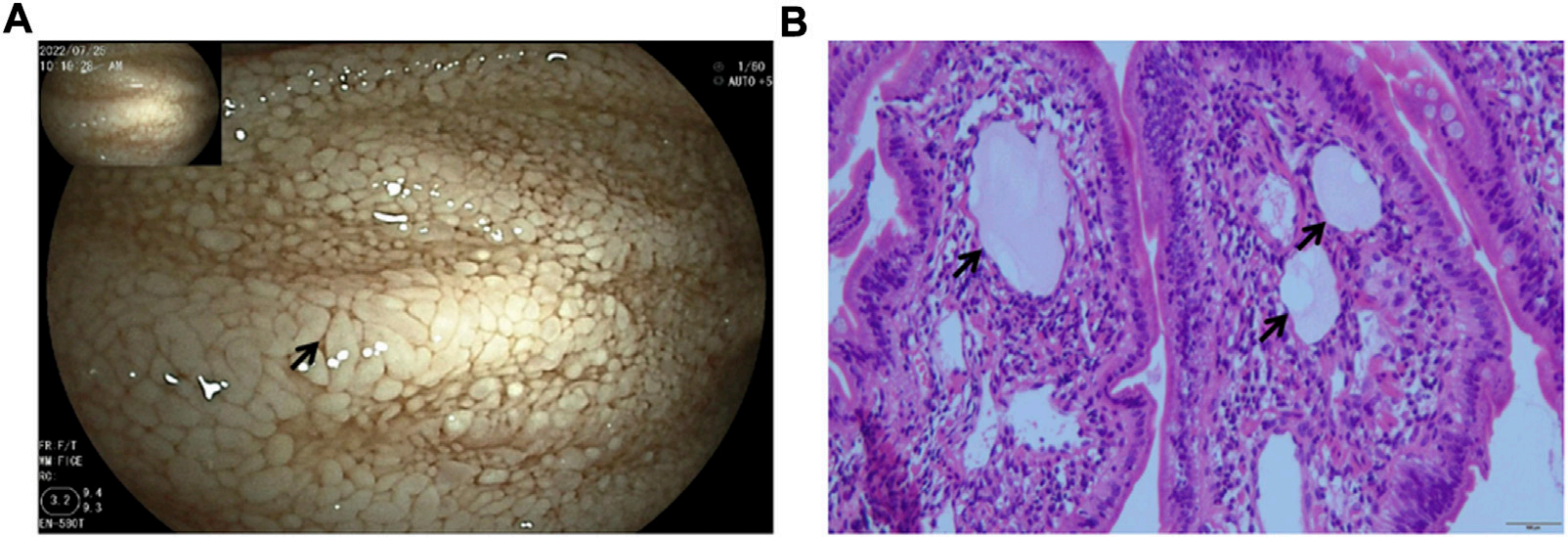
Gastroduodenoscopy images. **(A)** Scattered white spots and white villi covering the mucosa (arrowheads). **(B)** Markedly dilated lymphatic vessels (arrowheads) by the small bowel biopsy.

We next investigated the cause of hypoalbuminemia in the patient. The patient’s diet and liver function were normal, and the protein intake and synthesis were normal, suggesting that the hypoproteinemia may be caused by protein loss. The routine urine tests and urine protein were normal, indicating potential PLE. With the help of the digestive department, we completed a small bowel examination (fasting, bowel preparation until the stool becomes watery, tracheal intubation and general anesthesia are required due to the patient’s condition). Gastroduodenoscopy showed rough duodenal and jejunal segment mucosa and swollen and thickened villi with different sizes, and it also showed diffuse white granule-like changes and disorderly arranged villi. The intestinal fluid in the jejunal cavity was milky white. Lesions were present in the duodenal segment and the middle and upper segments of the jejunum. The small intestinal mucosa in the ileal segment was swollen with thickened and shortened villi, and a few white granules were present at the top. In the small intestinal mucosa, the villi were arranged in an orderly manner, and no obvious ulcers, proliferative foci or bleeding foci were found. These results suggested that the lymphatic vessels were dilated (Figure 1).

**FIGURE 2:**
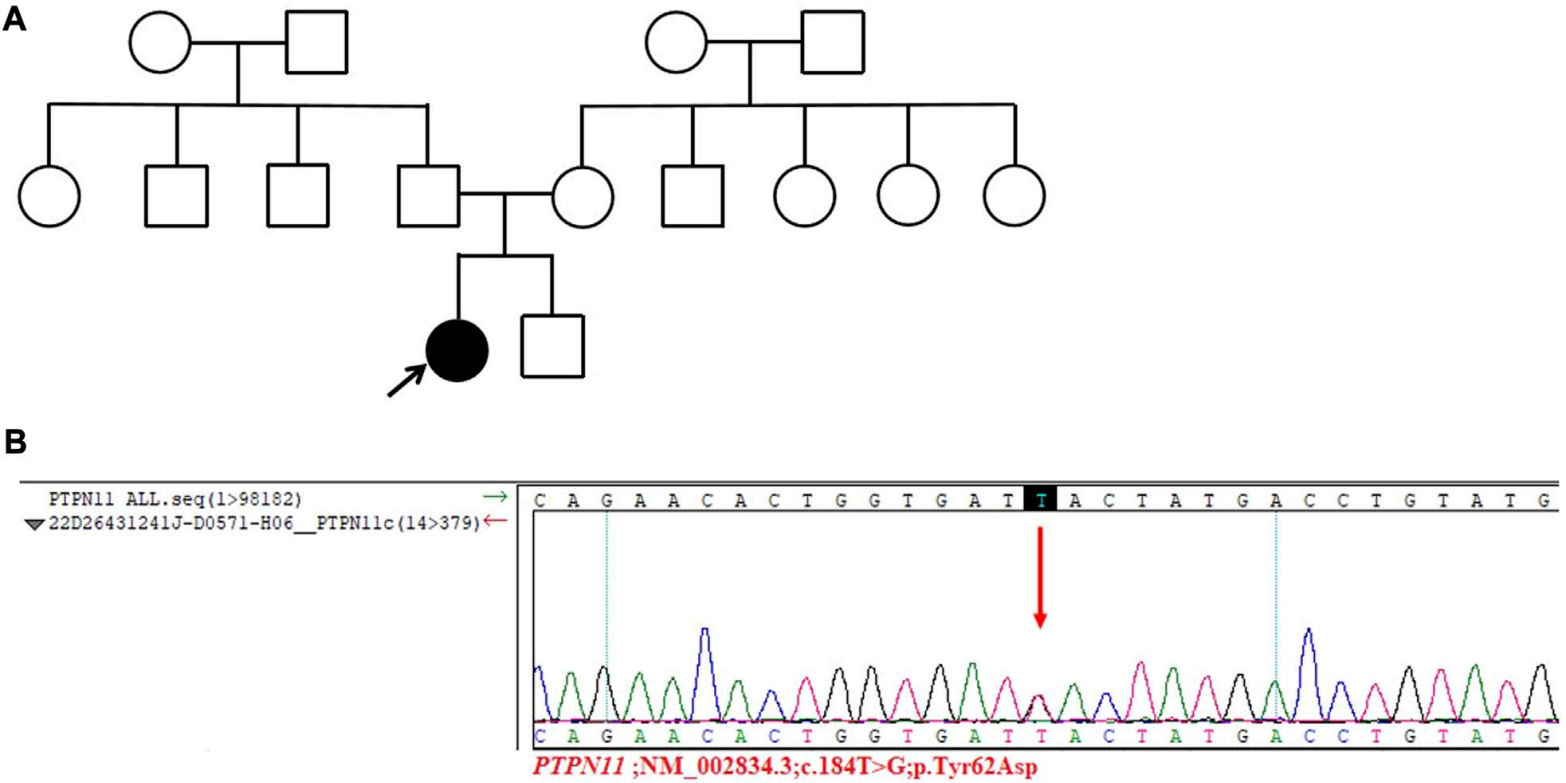
Results of the whole exome test. **(A)** Pedigree of the patient’s family. The patient’s family is represented in black, and the arrow represents the proband, which is the patient discussed in this article. **(B)** Sanger sequencing of the PTPN11 gene in the patient. A c.184T˃G missense mutation was found in the PTPN11 gene (p.Tyr62Asp) of the patient.

The diagnosis of Turner syndrome cannot be excluded based on the patient’s clinical signs. However, the chromosomal analysis results indicate 46, XX, which is inconsistent with Turner syndrome. To make a clear diagnosis, with the consent of the patient and herfamily, a whole exome test was performed. No mutation was detected in the patient’s mother and brother. The gene mutation results of the patient indicated a c.184T>G missense mutation (p.Tyr62Asp) in the PTPN11 gene (NM_002834.3) (Figure 2). Therefore, the patient was diagnosed with NS.

After discharge, the patient was treated with calcium carbonate D3 tablets (600 mg, twice a day)and ferrous succinate sustained-release tablets (one tablet, once a day). The patient was directed to consume a diet rich in medium-chain triglycerides (MCT), limit the intake of long-chain fatty acids, and increase their consumption of high-quality protein. Due to the advantages of MCT over long-chain fatty acids, including easier digestion, efficient energy supply, and reduced intestinal burden, they are suitable for patients with intestinal lymphatic dilation.

## Discussion

NS is usually accompanied with lymphodysplasia (Nistal et al., 1984; Ogata et al., 2003; Tartaglia et al., 2010). Up to 20% of NS patients have lymphedema secondary to lymphodysplasia (Ho et al., 2003). A previous cross-sectional cohort study comprised of 35 patients with NS has reported that the prevalence of lymphedema is 49% and that the lymphedema is chronic and intermittent, mainly occurring in the limbs (Smpokou et al., 2012). Lymphedema associated with NS can occur at all ages, but it usually occurs at birth (Witt et al., 1987). Some features of NS, such as webbed neck, low hanging ears, low hairline and ptosis, may be related to intrauterine edema (Opitz, 1986), whereas intestinal lymphangiectasia usually occurs later (Herzog et al., 1976).

PLE is a rare disorder of gastrointestinal protein loss caused by a variety of diseases (Mizuochi et al., 2015). Intestinal lymphangiectasia is one of the causes of PLE (Mizuochi et al., 2015), and it is associated with intestinal loss of serum protein due to nonspecific expansion of intestinal mucosal lymphatic vessels, which is manifested as hypoproteinemia, hypogammaglobulinemia, peripheral blood edema and lymphopenia (Herzog et al., 1976). This change of intestinal lymphatic vessels may be caused by secondary reasons, such as poor development or obstruction of lymphatic vessels, which may be observed in various congenital and acquired diseases. NS may be accompanied by exaggerated intestinal lymphatic vessels and congenital heart disease, which may lead to refractory PLE.

A comprehensive literature search of the PubMed database from 1972 to 2022 using the “Noonan syndrome” and “PLE” search terms revealed only 11 reported cases (Table 2).

**TABLE 2:** Summary of all patients with NS-associated PLE identified in the literature (1972-2022).

Cased	Sex	The onset of NS (yr)	The onset of PLE (yr)	Symptoms	Cardiac disorder	Transnodal lymphangiography	Treatments	Gene	Variant	Protein change	Follow-up
Matsumoto et al. (2015)	female	17	17	No	HCM	Absent thoracic duct; abdominal collateral lymphatics and bilateral iliac lymphangiectasia	Steroid therapy (1 mg/kg/d) Low-fat, protein-rich diet supplemented with medium chain triglycerides	Not provided			Relieved
Mizuochi et al. (2015)	female	1.5	8	Edema, Abdominal pain, diarrhea	ASD PVS	Not provided	Spironolactone (2.5 mg/kg/d) Furosemide (2.0 mg/kg/d)	Not provided			Relieved
Keberle et al. (2000)	male	19	21	Tibial edema Clubbing	Fallot`s tetralogy	Intestinal protein loss predominantly in the ileum	Low-fat, protein rich diet, medium chain triglycerides	Not provided			Relieved
Herzog et al. (1976)	female	0.9	15	Ankle swelling	ASD PVS	Hypoplasia of the lymphatics of the lower extremity and multiple ectatic lymph vessels in the mediastinal area and right supraclavicular area	Medium-chain triglyceride diet	Not provided			Relieved
Vallet et al. (1972)	Male	0.3	6	Diarrhea Anasarca, chylorrhea from the inguinal skin	PVS	Not provided	Medium-chain trigIycerides and a low-fat diet	Not provided			Died
Joyce et al. (2016)	male	26	27	genital swelling, bilateral lower limb lymphoedema	hypertrophic cardiomyopathy	Lymph reflflux/rerouting, bilateral popliteal LN, contrast in scrotum, multiple widened channels	low fat, high-protein diet	BRAF	c.770A>G	p.Gln257Arg	Not provided
Dori et al. (2020)	female			bleed	ventricular septal defect	unavailable	MEK inhibition	SOS1	c.2536G>A	p.E846K	Relieved
Kleimeier et al. (2022)	male	27	27	unavailable	unavailable	unavailable	unavailable	SOS1	c.1277A>C	p.Gln426Pro	Not provided
Wang et al. (2020)	female	7	30	Exrtimitis edema	Fllot`s tetralogy	Lymphangiectasis and bilateral widening of the venous angle in the mediastinum and small intestine	Low-fat, medium chain triglycerides	PTPN11	c.A922G	p.N308D	Relieved
Hasegawa et al. (2009)	male	6	13	edema of his lower abdomen and hydrocele testis	ASD PVS	Protein loss from the small intestine	Albumin (2.5 g) Growth hormone	Not provided			Relieved
Our case	female	19	19	edema of both lower limbs	ASD		Low-fat, medium chain triglycerides	PTPN11	c.184T>G	Tyr62Asp	Relieved

A patient with NS combined with intestinal lymphangiectasia was first reported in 1972 (Vallet et al., 1972), and there have been relevant reports since then (Herzog et al., 1976). There is a certain relationship between NS and lymphangiectasia, but there are not many cases of NS with lymphangiectasia. The severity of intestinal lymphatic dilatation may be the cause of chronic malabsorption and growth retardation in patients (Vallet et al., 1972). Via endoscopy, lymphangiectasia shows white dilated villi in the duodenum (Kumar et al., 2021). The Noonan Syndrome Research Group of the University of London studied 112 patients with NS, and they did not detect lymphangiectasia in these patients (Shaw et al., 2007).

A Ras/MAPK pathway gene mutation is a candidate gene for NS (Razzaque et al., 2007; Roberts et al., 2007). The MAPK pathway is activated by vascular endothelial growth factor receptor 3 (VEGFR3), which is an important component of lymphangiogenesis (Tammela et al., 2005). Although the genotype-phenotype correlation of NS is not exact, the severity of lymphangiogenesis disorders may vary depending on gene mutations. In addition, the severity of lymphangiogenesis disorders may lead to different clinical symptoms of NS, including asymptomatic, only lymphedema or both lymphedema and PLE as well as early or late onset in time (Hasegawa et al., 2009). Some studies have found that patients with neuroblastoma have mutations in the PTPN11 gene and that these patients also have PLE (Obasaju et al., 2018). The PTPN11 gene is the most common pathogenic gene of NS, accounting for 30%–60% of NS cases. The PTPN11 gene is located on chromosome 12q24, and it contains 15 exons and 14 introns with a total length of 91,182 bp (Athota et al., 2020). The PTPN11 gene encodes the SHP2 protein tyrosine phosphatase, which contains three domains, namely, N-SH2, C-SH2, and PTP. The PTP domain contains phosphorylation active sites, and its activity is inhibited by the N-SH2 domain (Tajan et al., 2015). Approximately 50% of NS cases are caused by the missense variation of PTPN11 gene function acquisition. These variations are mainly distributed in the N-SH2 or PTP domain, rendering the inhibition of the N-SH2 domain ineffective. The PTP domain is also activated without phosphorylation via ligand binding, and upregulation of the Ras/MAPK signaling pathway leads to NS (Tajan et al., 2015). Eleven cases of PLE in NS have been recorded, including two cases associated with a germline PTPN11 mutation, involving the c.A922G and c.181G>A sites. The present case also involved a c.184T>G missense mutation of the PTPNT11 gene (p.Tyr62Asp), and PLE was the main clinical manifestation. It is unclear whether the mutation in the PTPN11 gene is more likely to cause PLE. The most common features of patients with NS due to PTPN11 mutations include heart defects (74%), low ear position (80%), low posterior hairline (68%), lower strabismus blepharoplasty (68%), cryptorchidism (94% of boys) and short stature (93%) (Zandrino et al., 2003; Yoshida et al., 2004; Sznajer et al., 2007). An infant with pulmonary lymphangiectasia (PL) was diagnosed with NS by genomic DNA sequence analysis after death because he had a G503R heterozygous mutation in the PTPN11 gene (Mathur et al., 2014). Pieper et al. diagnosed nine patients with NS and lymphatic abnormalities through dynamic contrast enhanced magnetic resonance (MR) lymphography, and among them, five patients had PTPN11 gene mutation (Pieper et al., 2022).

Japanese scholars have investigated the PTPN11 gene in 21 patients with NS; they identified 6 different heterozygous missense mutations in 7 cases (Asp61Gly, Tyr63Cys, Ala72Ser, Thr73Ile, Phe285Ser, and Asn308Asp) (Kosaki et al., 2002), but there was no report of PLE in these cases. It is currently believed that mutations in the PTPN11 gene may lead to relatively rare features of NS, such as sensory deafness and hemorrhagic diathesis (Kosaki et al., 2002). However, it remains unknown whether mutation of the PTPN11 gene is related to PLE, thereby warranting additional studies for confirmation.

In a previous case report for one patient with NS, an absent thoracic duct, abdominal collateral lymphatics and bilateral iliac lymphangiectasia were identified via lymphangiography (Matsumoto et al., 2015). It has also been reported that pedal lymphography and postlymphangiographic CT can be used to diagnose and study PLE (Keberle et al., 2000).

Dietary therapy, including low-fat diet and medium-chain triglyceride diet, is currently the cornerstone of lymphangiectasia therapy (Kumar et al., 2021). The medium-chain triglyceride diet also has a good effect on patients with NS complicated by exaggerated intestinal lymphatic vessels (Holt, 1964; Herzog et al., 1976). The medium-chain triglyceride diet has been shown to significantly improve hypoproteinemia, hypocalcemia and hypogammaglobulinemia. A previous study has reported that a patient with NS and congenital heart disease treated with standard doses of spironolactone and furosemide showed successful treatment of refractory PLE (Mizuochi et al., 2015), suggesting that diuretics effectively treat refractory PLE in patients with NS, but the specific physiological mechanism is unclear. Because the etiology of NS is the activation of the Ras/MAPK pathway, treatment with Ras/MAPK inhibitors may be feasible. At present, MEK inhibitors have been successfully used in patients with NS and severe lymphatic dysplasia (Li et al., 2019; Dori et al., 2020).

In terms of clinical presentation, consistent with our case, patients with protein-losing enteropathy (PLE) due to intestinal lymphangiectasia often exhibit hypoalbuminemia, particularly a significant decrease in albumin levels. They may also present with electrolyte disturbances such as severe hypocalcemia (Dori et al., 2020; Wang et al., 2020). When encountering cases of unexplained hypoalbuminemia in the absence of inadequate intake, non-protein synthesis disorders, or non-renal loss of protein, it is necessary to consider PLE as a potential diagnosis.

In conclusion, NS accompanied with PLE is not common, but asymptomatic hypoproteinemia attributable to PLE may be more common in patients with NS than previously thought. Therefore, when patients with NS have unexplained hypoproteinemia and hypoelectrolytes, they should be further evaluated for the presence of PLE, especially in patients with PTPN11 gene mutation.

## Data Availability

The original contributions presented in the study are included in the article/supplementary material, further inquiries can be directed to the corresponding authors.

## Abbreviations

NS, noonan syndrome; PLE, protein- loss enteropathy; MAPK, mitogen activated protein kinase; PTPN11, protein tyrosine phosphatase non-receptor type 11; SOS1, son of sevenless homolog 1; RAF1, raf-1 proto-oncogene, serine/threonine kinase; NRAS, neuroblastoma RAS viral oncogene homolog; SHOC2, soc-2 suppressor of clear homolog; CCEB1, collagen and calcium binding EGF domains 1; FAT4, FAT tumor suppressor homolog 4; PIEZO1, piezo type mechanosensitive ion channel component 1; FOXC2, forkhead box C2; CD55, complement decay-accelerating factor; VEGFR3, vascular endothelial growth factor receptor 3; PL, pulmonary lymphangiectasia; MCT, medium-chain triglycerides.
